# Development of a Minimum Data Set Registry for Chronic Venous Insufficiency of the Lower Limbs

**DOI:** 10.3390/jcm8111779

**Published:** 2019-10-24

**Authors:** Erica Homs-Romero, Angel Romero-Collado

**Affiliations:** 1 Figueres Basic Healthcare Area (Àrea Bàsica de Salut de Figueres), Catalan Health Institute (Institut Català de la Salut), C/Tramuntana 2, 17600 Figueres (Girona), Spain; ericahr@gmail.com; 2 Nursing Department, University of Girona, C/Emili Grahit 77, 17071 Girona, Spain

**Keywords:** diagnosis, information management, signs and symptoms, venous insufficiency, venous ulcer

## Abstract

The purpose of this study was to develop a minimum data set (MDS) registry for the prevention, diagnosis and treatment of chronic venous insufficiency (CVI) of the lower limbs. We designed the instrument in two phases, comprising a literature review and an e-Delphi study to validate the content. We obtained a total of 39 documents that we used to develop a registry with 125 items grouped in 7 categories, as follows: Patient examination, venous disease assessment methods, diagnostic tests to confirm the disease, ulcer assessment, treatments to manage the disease at all its stages, patient quality of life, and patient health education. The instrument content was validated by 25 experts, 88% of whom were primary healthcare and hospital nurses and 84% had more than 10 years’ experience in wound care. Using a two-round Delphi approach, we reduced the number of items in the MDS-CVI to 106 items. The categories remained unchanged. We developed an MDS for CVI with seven categories to assist healthcare professionals in the prevention, early detection, and treatment history of CVI. This tool will allow the creation of a registry in the primary care setting to monitor the venous health state of the population.

## 1. Introduction

Chronic venous disease (CVD) of the lower limbs is a health problem with high prevalence and gradual progression. Developed countries are starting to manage this disease at early stages, in an attempt to prevent complications such as ulcers, when the human and economic burden is very heavy [1,2].

The evidence shows that lower limb venous disease can be staged by means of comprehensive history-taking that covers the classic signs and symptoms of venous disease, and correct Clinical, Etiological, Anatomical, and Pathophysiological (CEAP) classification [3,4]. The CEAP classification consensus document was published by the American Venous Forum in 1994 and was last updated in 2004. The aim of this instrument is to improve scientific communication when describing venous disease.

The CEAP clinical classification ranges from C0 (no visible or palpable signs of venous disease) to C6 (active venous ulcer). The system permits a patient’s status to be classified by the presence of signs such as reticular veins, oedema and trophic skin changes. These signs are accompanied by symptoms such as pain, heaviness, burning sensation, cramps, and pruritus [5]. The quality of life of individuals with CVD is drastically reduced as the disease advances [2].

Chronic venous insufficiency (CVI), defined as CEAP clinical classes C3–C6, affects 5% of the population, and an estimated 1–2% have a leg ulcer at some stage in their lives [6,7]. Active ulcers are responsible for the main financial impact of the disease process. The cost of caring for patients with CVI is estimated at 600–900 million euros in western Europe, accounting for 2% of healthcare expenditure. The estimated mean direct cost of each ulcer is €9000, representing 90% of the total CVI bill. This figure includes the cost of human resources (doctors and nurses), material for dressings, and hospital stays. Another less visible component is the indirect cost of CVI, which includes patients’ and relatives’ travel expenses, time off work, and even disability [5,8].

In the primary healthcare (PHC) setting, the clinical component (C) of CVD can be classified by means of patient questioning, thorough history taking, and a physical examination with the patient in a standing position, to observe dilated veins and skin abnormalities. The Doppler-assisted ankle-brachial index (ABI) must also always be calculated to make an accurate diagnosis and rule out peripheral arterial disease [6].

Venous disease prevention, diagnosis, and most treatment can take place in the primary care setting, but healthcare professionals must be appropriately trained and have the tools to provide this care. Patients may benefit from surgery at more advanced stages and will therefore need to be referred to the angiology or vascular surgery department [9].

Despite clear scientific evidence showing that the gold standard of CVI prevention and treatment is lower limb compression by means of bandaging, stockings, and other devices, in clinical practice, these measures are rarely implemented [3]. In fact, as many as 90% of patients with CVI receive no treatment whatsoever [10]. The literature describes several factors that might explain the low uptake of compression treatment, including a lack of awareness and skills among healthcare professionals [11,12].

A minimum data set (MDS) is a set of clearly defined items concerning a specific issue. MDSs have been shown to be effective in the prevention and early detection of different health problems, and to help guide their treatment [13,14]. A MDS permits interventions to be planned and followed up over time, and identifies which minimum quality indicators should be implemented [15]. The purpose of this study was to develop a MDS registry for CVI (MDS-CVI) of the lower limbs.

## 2. Methods

The instrument was designed in two phases, as follows: A literature review and an e-Delphi study with content validation by an expert panel.

### 2.1. Phase 1. Literature Review

We performed a literature review to define the MDS-CVI parameters. In December 2015, we carried out a literature search of keywords in MEDLINE (via PubMed), Cumulative Index to Nursing and Allied Health Literature (CINAHL), Scopus, and Cochrane Library Plus.

In PubMed and SCOPUS, we used the Medical Subject Headings (MeSH) terms ‘Diagnosis’, ‘Signs and Symptoms’, and ‘Venous Insufficiency’. In the CINAHL database, we used the MeSH terms ‘Diagnosis’ and ‘Venous insufficiency chronic’. The Boolean operator “AND” was used in all searches. In the Cochrane Library Plus database, we used the term “Venous Insufficiency”.

We used the Google search engine to find clinical practice guidelines and scientific society publications related to chronic wound care.

Inclusion criteria were language (English or Spanish), publication date (2011 or later), pathology (CVI of the lower limbs, venous ulcers), and treatment (of CVI of the lower limbs).

Two researchers analyzed the articles independently to identify concepts related to the prevention, diagnosis, or treatment of venous disease of the lower limbs. Then, they reached a consensus on the definitive items.

### 2.2. Phase 2. e-Delphi Study

We used an e-variant of the original Delphi study, which gathers experts’ opinions to reach a consensus on a complex issue. The e-Delphi format was used to obtain data through an online platform [16]. The purpose of the study was for wound care experts to assess the validity of the MDS-CVI content obtained through the literature review.

#### 2.2.1. Sample

To create the expert panel, we contacted the six leading Spanish scientific societies for vascular diseases and wounds, as follows: Grupo Nacional para el Estudio y Asesoramiento en Úlceras por Presión y Heridas Crónicas (National Advisory Study Group for Pressure Ulcers and Chronic Wounds) (GNEAUPP), Asociación Nacional de Enfermería Dermatológica e Investigación del Deterioro de la Integridad Cutánea (National Association of Dermatology Nursing and Research into Deterioration of Skin Integrity) (ANEDIDIC), Sociedad Gallega de Heridas (Galician Society for Wounds) (SGH), Asociación Española de Enfermería Vascular y Heridas (Spanish Association for Vascular Nursing and Wounds) (AEEVH), Sociedad Española de Heridas (Spanish Society for Wounds) (SEHER), and the Sociedad Española de Angiología y Cirugía vascular (Spanish Society for Angiology and Vascular Surgery) (SEACV). These societies wrote to their members to describe the study objectives and methods, and provided an email address where members could request more information about the study with a view to participating in the panel.

#### 2.2.2. Ethical Considerations

The study protocol was reviewed and approved by The Foundation University Institute for Primary Health Care Research Jordi Gol i Gurina (IDIAPJGol), under code P17/030.

All participants were required to sign a privacy agreement and study participation consent form before joining the expert panel.

#### 2.2.3. Data Collection

The experts participated in two rounds by completing a questionnaire drawn up on the Google Forms platform.

#### 2.2.4. e-Delphi Round 1

The first round, carried out in April 2017, contained the 125 items from the literature review, grouped into seven categories. The experts had to consider the suitability of the items for inclusion in the MDS-CVI and grade them on a scale of 1 to 5, where 1 was very unsuitable and 5 was very suitable.

The experts were informed that consensus would be established for items with a mean score of 4. A high consensus was defined as ≥72% of experts scoring ≥4 for an item, which is slightly higher than the 70% recommended by some authors [17]. Items that achieved this level of consensus were marked as definitive and excluded from the second round. Items with a mean score between 3.5 and <4 and a consensus of 50% to 72% were reviewed in the next round. Items with a mean score of <3.5 and a consensus of <50% were deleted. The experts were allowed to suggest new items and categories.

#### 2.2.5. e-Delphi Round 2

In the second round, carried out in June 2017, the results from the first round were shared, new items proposed by the experts were added, and the method and criteria applied in the first round were repeated.

## 3. Results

### 3.1. Phase 1. Literature Review

**Figure 1:**
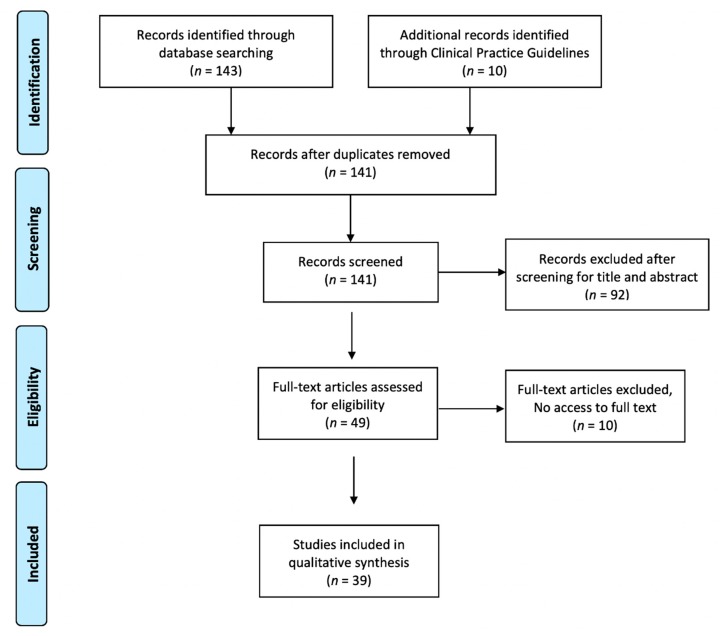
Flow diagram showing studies identified and selected.

A total of 153 articles were obtained from the literature search (Figure 1). After removal of duplicate articles, those not meeting the inclusion criteria and those we were unable to access, 39 articles were included in the analysis.

With these 39 documents, we developed an MDS for the prevention, diagnosis, and treatment of CVI, with a total of 125 items grouped into seven categories, as follows:

(1) Patient examination [3,4,5,6,18,19,20,21,22,23,24,25,26,27,28,29,30,31,32,33,34,35,36,37,38,39,40,41,42,43,44,45,46,47,48,49,50,51,52,53,54,55] (Table 1), with two sub-categories, as follows:
  (a) Risk factors, with 15 items covering personal circumstances that increase the likelihood of CVD. These items include age, sex, and family history of CVI.
  (b) Leg conditions, with 22 items related to the signs and symptoms of venous disease of the lower limbs such as cramps, heaviness, and varicose veins.
(2) Diagnostic studies [6,21,23,26,27,28,29,30,31,32,33,34,35,36,38,39,40,42,43,44,47,49,50,51,52,53] defining venous disease (Table 2), with eleven items describing existing diagnostic tests. These tests include continuous wave-Doppler and duplex ultrasound.
(3) Scoring and classification systems [6,19,21,22,23,24,26,29,33,38,42,43,44,45,46,47,48,49,50,51] with three items. Venous disease scoring and classification systems consisted of the Villalta scale, the Venous Clinical Severity Score (VCSS) and the CEAP.
(4) Ulcer examination [30,31,33,34,39,42] with seven items to describe ulcers, including photography, signs of infection, and pain.
(5) Different treatments at the various stages of venous disease (Table 3). This category has four sub-categories, as follows:
  (a) Compression therapy [6,19,21,26,27,29,30,31,32,33,34,35,36,38,39,41,42,44,46,47,49,50,51,53] with seven items covering different limb compression methods, including the Unna boot, graduated compression hosiery, and the multi-layer compression bandage system.
  (b) Drug treatment [6,21,29,33,35,36,39,42,43,44,46,52,53] with ten items related to recommended drugs in venous disease, such as oral anticoagulants, flavonoids/phlebotonics, and pentoxyphylline.
  (c) Surgical treatment [6,19,21,23,26,28,40,43,44,45,47,49,50,53,54,55] with nine items, including mechanochemical endovenous ablation (MOCA) and endovenous thermal ablation (EVTA).
  (d) Venous ulcer treatment [4,6,25,29,30,31,33,34,39,44] with 20 items. Treatments range from ulcer cleansing to ultrasound therapy or vacuum-assisted closure (VAC).
(6) Patient quality of life [6,21,27,40,44,47,49,56] (Table 4), with five scales to assess patients’ quality of life at different stages of venous disease, such as the Chronic Venous Insufficiency Quality of Life Questionnaire (CIVIQ) for people with CVI and the Charing Cross Venous Ulcer Questionnaire for individuals with venous ulcers.
(7) Health education [29,30,31,33,34,42,51] with 16 items, including recommendations to prevent complications and improve venous return, such as elevating the legs when resting, avoiding tight clothing, and taking regular exercise.

### 3.2. Phase 2. e-Delphi Study

A total of 25 experts participated in both rounds, of whom 72% were men, 88% were nurses, and 12% were doctors specialized in vascular disease. Most worked in primary healthcare or hospital settings, and combined this work with university teaching (72%). A total of 84% had more than 10 years of experience in wound care.

In the first round, the experts added 11 items (see items without literature citation in the tables) and at the end of that round, 10 items were deleted, 25 were moved to the next round, and 90 were marked as definitive.

**Figure 2:**
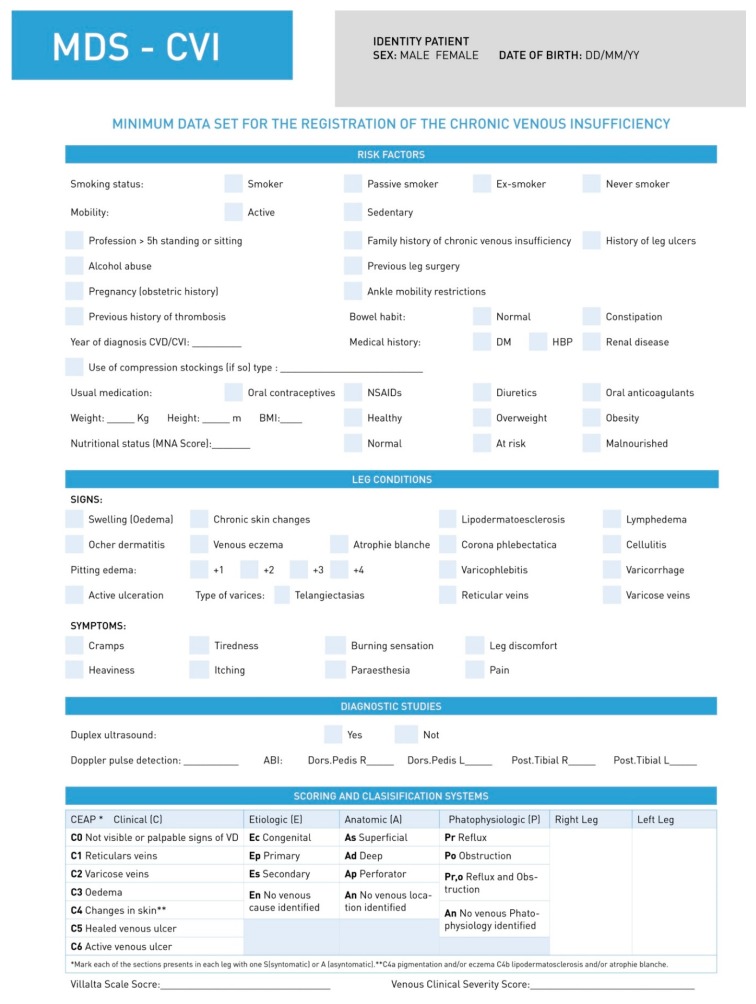
First page of the MSD-CVI.

**Figure 3:**
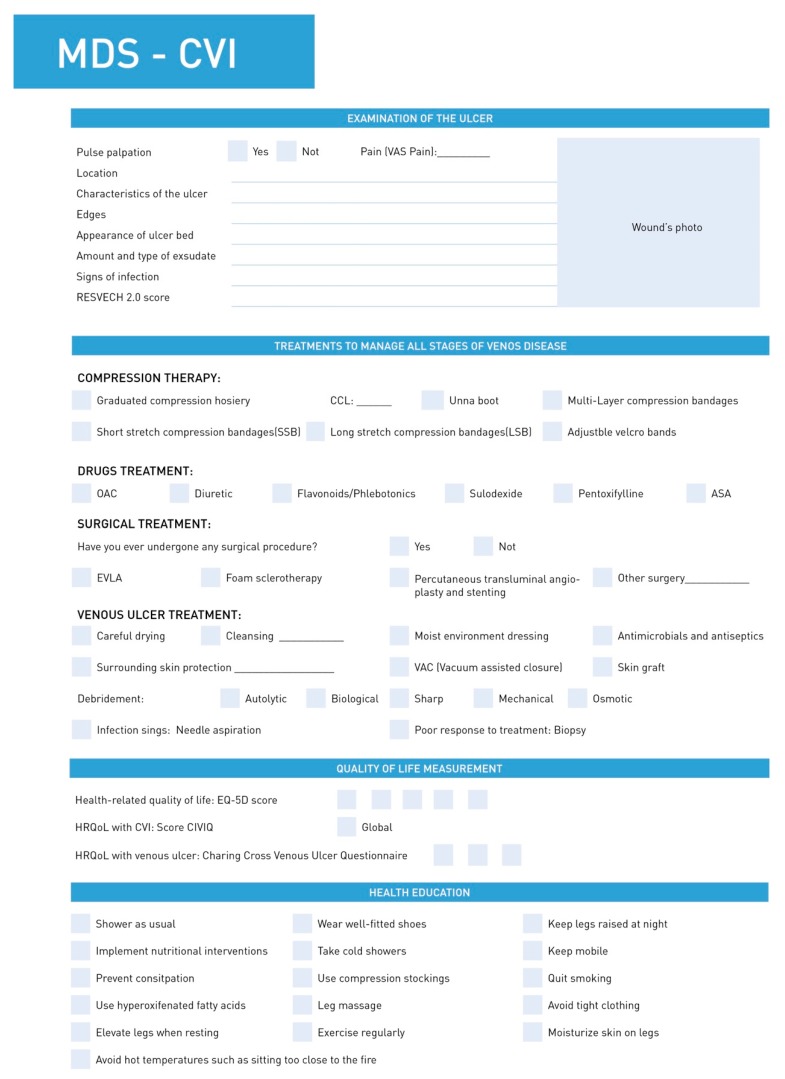
Second page of the MSD-CVI.

In the second round, the experts added no further items. At the end of the round, 20 items were deleted and 15 were accepted. The resulting MDS-CVI had a total of 106 items and 7 categories (Table 1, Table 2 and Table 3, Figure 2 and Figure 3).

## 4. Discussion

The MDS-CVI is primarily a data-collection tool. However, when completing the registry, healthcare professionals are reminded of important actions that can be carried out in people with risk factors such as older age [3,6,18,19,20,21,22,23,24,25], female sex [6,21,22,24,26,28], and obesity [6,19,21,22,23,26,28,29,31,33,35,42], which increase their likelihood of having CVD [6]. Activities to promote health, prevent CVD, and diagnose it at earlier stages will help halt or delay disease progress. Healthcare professionals, and those working in primary health in particular, should aim to educate at-risk patients to lead a healthy lifestyle and use compression stockings.

Due to increased awareness of CVD, the tendency is generally for earlier diagnosis and treatment. However, in some countries, the disease is not detected until more advanced stages. There are gaps in healthcare professionals’ knowledge of venous leg ulcer physiology and its healing process [11], partly due to a lack of training at a degree level [12]. By applying and incorporating this MDS-CVI in patients’ health records, healthcare professionals will find it easier to monitor the disease course at every stage [6]. Above all, they should follow the recommendations to ensure correct diagnosis and treatment.

The CEAP classification system is a very easy method to classify venous disease and reach a reliable diagnosis of CVD/CVI in the population. The clinical part of the system can be obtained simply by observing a patient’s legs in the primary care setting. It is estimated that 80% of the population have the mildest level of symptoms (C1–C2, spider and varicose veins), while 5% have the most advanced stages (C3–C6) [6]. Implementation of this evidence-based MDS-CVI would result in more reliable data collection and facilitate monitoring of a specific population to observe disease progression, the treatments used, and their effectiveness [13,14]. With the existing level of evidence of the importance of therapeutic compression of the lower limbs, it is unacceptable that 90% of patients with CVI in Turkey [10] and 54% of patients with venous ulcers in Spain [57] are not given compression stockings. The MDS-CVI will also permit health managers to plan interventions according to the venous state of the population and identify which quality indicators should be applied [17].

People with CVI have a poor quality of life [58]. It is therefore important to determine how the venous disease affects each individual. Specific instruments are available to measure quality of life in these patients, such as the Aberdeen Varicose Vein Questionnaire (AVVQ) [21,40] or the Chronic Venous Insufficiency Quality of Life Questionnaire (CIVIC) [27] for patients with CVI, and the Charing Cross Venous Ulcer Questionnaire [56] for patients with venous ulcers. The instruments are valid for only certain languages and cultures [59] and they therefore need to be adapted to be effective.

Non-pharmacological measures are essential in the prevention and adjuvant therapy of CVD and healthcare professionals should therefore be aware of their existence and use them in their clinical practice. Recommendations such as weight loss [30,31,33,34,42] or taking regular exercise [29,33,34,42] will help venous return and delay symptom progression.

The MDS for CVI establishes minimum quality care criteria and can help to guide in the purchase of necessary services.

## 5. Limitations

One limitation of the review is that we were unable to access the full text of 10 articles that appeared in our literature search, although the addition of the 10 clinical practice guidelines helped overcome this limitation, at least in part.

In addition, all participants were from Spain, which may have given more or less importance to certain interventions and/or instruments than others. For example, the Aberdeen Varicose Vein Questionnaire was excluded from our study because no Spanish-language validation is available. On the contrary, the RESVECH 2.0 scale—an instrument that assesses chronic wound progression—was included but has no English-language validation [60]. Nevertheless, the literature review and the details of the items that were added and excluded by the experts make it easy to view the items that were assessed, and they can be easily adapted according to the needs of each health system.

Another limitation of the study is that most participants were nurses, and this may explain the elimination of some items from the e-Delphi data set related to non-nursing procedures, such as radiofrequency ablation.

## 6. Conclusions

We have developed a MDS for CVI with seven categories and 106 items to assist healthcare professionals in the prevention, early detection, and treatment history of CVI. This MDS-CVI also enables the creation of a population-based registry in the primary care setting to monitor the venous health state of the population, the pathological evolution over time, characteristics of the population, attention provided, and the distribution of health resources destined or necessary for the complete care of the person suffering from CVI.

## Figures and Tables

**Table 1:** Items related to risk factors and patient’s leg conditions.

Risk Factors					
	Mean Round 1	Exp ≥ 4 *n* (%)	Mean Round 2	Exp ≥ 4 *n* (%)	Final Decision
Usual medication [29,30]: Diuretics [36], Nonsteroidal anti-inflammatory drugs (NSAIDs) [36], Oral anticoagulants [38,40], Oral contraceptives [6,27,28,33,35]	4.76	25 (100)			Kept
Mobility: Sedentary [31,34,42]	4.76	24 (96)			Kept
Gender [6,21,22,24,26,27,28]	4.76	23 (93)			Kept
Obesity (Body mass index ≥ 30) [6,19,21,22,23,26,28,29,31,33,35,42]	4.72	25 (100)			Kept
Clinical history [30,34]: Diabetes Mellitus (DM) [26,35], Arterial Hypertension (HTA) [35,36]	4.72	24 (96)			Kept
Family history of chronic venous insufficiency [6,22,23,24,28,31,34]	4.72	24 (96)			Kept
Job [23,24,28,38,39,40,41]	4.68	24 (96)			Kept
Age [3,6,18,19,20,21,22,23,24,25]	4.6	23 (92)			Kept
Renal disease [26,35,36,40]	4.52	22 (88)			Kept
Smoking status [21,26,28]	4.52	21 (84)			Kept
Ankle mobility restrictions [29,30,33,34]	4.36	22 (88)			Kept
Nutritional status [31,34]	4.28	21 (84)			Kept
Bowel habit [28]	4.20	20 (80)			Kept
Pregnancy (obstetric history) [22,23,26,27,28,34,38,40,43]	4.12	20 (80)			Kept
Ethnicity [6,21,26,28]	3.56	15 (52)	3.44	15 (60)	Removed
History of leg ulcers			4.88	25 (100)	Kept
Previous history of thrombosis			4.84	25 (100)	Kept
Use of compression stockings			4.68	23 (92)	Kept
Previous surgical background of the legs			4.48	23 (92)	Kept
Year of diagnosis CVD/CVI			4.24	20 (80)	Kept
Harmful alcohol consumption			4.08	20 (80)	Kept
**Leg conditions: Symptoms**					
Heaviness [6,21,24,27,31,33,44,46,47,48,49]	4.80	25 (100)			Kept
Itching [6,21,23,31,33,34,35,36,37,44,46,47,49]	4.60	24 (96)			Kept
Pain [6,21,23,24,25,26,27,31,33,34,35,40,44,46,47,49,51,52]	4.60	23 (92)			Kept
Cramps [6,23,30,31,33,35,44,47]	4.52	24 (96)			Kept
Burning sensation [21,23,44,45,46]	4.48	23 (92)			Kept
Paraesthesia [46]	4.44	22 (88)			Kept
Discomfort legs [38,44,48]	4.32	22 (88)			Kept
Tiredness [21,41,46,49]	4.24	20 (80)			Kept
**Leg conditions: Signs**					
Active ulceration [19,21,26,27,28,33,35,42,44]	4.96	25 (100)			Kept
Swelling (Oedema) [21,23,26,27,28,29,30,31,32,33,34,35,36,38,42,44,45,46,52,53,54]	4.96	25 (100)			Kept
Varicose veins [19,23,28,31,34,38,39,40,42,44,45,46,47,48,50,52,55]	4.92	25 (100)			Kept
Lipodermatosclerosis [28,32,34,35,39,44,46,51,53]	4.88	25 (100)			Kept
Venous eczema [23,28,29,30,31,32,34,35,42,44,46]	4.88	25 (100)			Kept
Atrophie blanche [28,31,33,34,39,42,46]	4.84	25 (100)			Kept
Telangiectasias [24,26,28,35,38,44,46]	4.80	25 (100)			Kept
Ocher dermatitis [33,42,44]	4.80	24 (96)			Kept
Chronic skin changes [6,21,30,31,34,39,40,44,46,49,52]	4.76	24 (96)			Kept
Corona phlebectatica [6,28]	4.68	24 (96)			Kept
Varicophlebitis [34]	4.68	24 (96)			Kept
Cellulitis [35]	4.60	23 (92)			Kept
Reticular veins [24,28,44]	4.60	23 (92)			Kept
Varicorrhage [21]	4.56	22 (88)			Kept
Pitting edema			4.76	24 (96)	Kept
Lymphedema			4.04	20 (80)	Kept

**Table 2:** Items related to diagnosis, scoring classifications systems, and examination of the ulcer.

Diagnostic Studies					
	Mean Round 1	Exp ≥ 4 *n* (%)	Mean Round 2	Exp ≥ 4 *n* (%)	Final Decision
Ankle brachial pressure index (ABPI) [6,29,30,31,33,34,35,39,42]	4.56	22 (88)			Kept
Duplex ultrasound [6,21,23,27,28,31,33,34,38,39,40,44,47,49,50,51]	4.44	22 (88)			Kept
D-dimer assay [35]	3.44	14 (56)	3.36	11 (44)	Removed
Trendelenburg test [28,31]	3.6	18 (72)	3.76	15 (60)	Removed
Perthes test [31]	3.56	16 (64)	3.76	16 (64)	Removed
Schwart test [33]	3.56	16 (64)	3.64	15 (60)	Removed
Continuous wave-doppler [6,21,26,30,31,32,33,35,36,40,43,44,47,50,53]	3.36	16 (64)			Removed
Air-Plethismography [6,32,33,34,44]	3.24	10 (40)			Removed
Venography [44,52,53]	3.08	10 (40)			Removed
Pulse oximetry [34,39]	3	10 (40)			Removed
Magnetic resonance [35,44,53]	2.92	9 (36)			Removed
Samuels maneuver			3.72	16 (64)	Removed
**Scoring and classification systems**					
CEAP classification of chronic venous disease [6,19,21,22,23,24,26,29,33,38,40,42,43,44,45,46,47,48,49,51]	4.80	24 (96)			Kept
Venous Clinical Severity Score (VCSS) [6,21,40,44,47,50]	4.60	22 (88)			Kept
Villalta score [6,44]	3.92	18 (72)	4.08	18 (72)	Kept
**Examination of the ulcer**					
Location [30,31,33,34,39,42]	5	25 (100)			Kept
Appearance of ulcer bed [30,31,33,34,39,42]	4.96	25 (100)			Kept
Characteristics of the ulcer [30,31,33,42]	4.88	25 (100)			Kept
Edges [33,34,39,42]	4.88	25 (100)			Kept
Pain [30,31,33,39]	4.88	25 (100)			Kept
Amount and type of exudate [30,34,42]	4.88	24 (96)			Kept
Signs of infection [34]	4.88	24 (96)			Kept
Leg pulses			4.64	23 (92)	Kept
RESVECH 2.0 score			4.60	25 (100)	Kept

**Table 3:** Items related to treatments to manage all stages of venous disease.

Compression Therapy					
	Mean Round 1	Exp ≥ 4 *n* (%)	Mean Round 2	Exp ≥ 4 *n* (%)	Final Decision
Graduated compression hosiery [19,21,26,27,30,32,33,34,35,38,39,41,42,44,46,49,50,51,53]	4.84	25 (100)			Kept
Multi-layer compression bandage system [6,19,29,30,33,39,42]	4.8	23 (92)			Kept
Long stretch compression bandages (LSB) [6,19,29,30,31,32,33,34,36,39,42,47,49]	4.6	24 (96)			Kept
Short stretch compression bandages (SSB) [6,19,29,30,31,33,34,39,42,47]	4.56	22 (88)			Kept
Adjustable Velcro bands [6]	4.52	24 (96)			Kept
Unna boot [6,29,31,39]	4.08	18 (72)			Kept
Pneumatic cuff compression [29,35,39,44]	3.72	15 (60)	3.52	15 (60)	Removed
**Drug treatment**					
Flavonoids/Phlebotonics [33,39,42,44,46]	4.28	20 (80)			Kept
Sulodexide [6]	4.28	20 (80)			Kept
Pentoxifylline [6,29,33,39,42,46]	4.08	18 (72)			Kept
Antibiotic [21,39]	3.96	15 (60)	3.80	17 (68)	Removed
Acetylsalicylic acid [6,39]	3.92	17 (68)	4.08	18 (72)	Kept
Diuretic [35,52]	3.88	16 (64)	4.08	20 (80)	Kept
Oral anticoagulants [35,43,44,53]	3.80	14 (56)	4.08	19 (76)	Kept
Gabapentin [36]	3.68	14 (56)	3.40	12 (48)	Removed
Horse chestnut extract [35,44,46]	3.40	11 (44)			Removed
**Herbal substances**					
Ruscus extract [44]	3.40	11 (44)			Removed
**Surgical treatment**					
Foam sclerotherapy [6,19,23,28,44,49,50]	4.16	19 (76)			Kept
Endovenous laser ablation (EVLA) [6,40,44,47,49]	4.04	19 (76)			Kept
Percutaneous transluminal angioplasty and stenting [53,54]	4.04	18 (72)			Kept
Radiofrequency ablation (RFA) [21,26,40,44,45,47,50]	3.96	17 (68)	3.92	17 (68)	Removed
Endovenous thermal ablation (EVTA) [6,21,23,44,50]	3.92	17 (68)	3.88	16 (64)	Removed
Ambulatory conservative haemodynamic management of varicose veins (CHIVA) [6,19,21,28,44,49,50,55]	3.88	17 (68)	3.92	16 (64)	Removed
Mechanochemical endovenous ablation (MOCA) [6,40,47,50]	3.84	15 (60)	3.84	17 (68)	Removed
Steam vein sclerosis (SVS) [43]	3.72	14 (56)	3.84	15 (60)	Removed
Cyanoacrylate embolization [21]	3.68	14 (56)	3.72	14 (56)	Removed
**Venous ulcer treatment**					
Cleansing [30,31,39]	4.80	23 (92)			Kept
Moist environment dressing [6,29,30,31,33,39,44]	4.76	24 (96)			Kept
Surrounding skin protection [33,39]	4.76	23 (92)			Kept
Autolytic debridement [29,31,39]	4.64	23 (92)			Kept
Sharp debridement [29,31,39]	4.64	23 (92)			Kept
Biological debridement [29,31,39]	4.52	23 (92)			Kept
Topical antimicrobials and antiseptics [29,30,33,39]	4.48	20 (80)			Kept
Mechanical debridement [29,31,39]	4.44	22 (88)			Kept
Vacuum assisted closure (VAC) [4,25,31,39]	4.28	76 (19)			Kept
Osmotic debridement [29,31,39]	4.12	18 (72)			Kept
Careful drying [30,31,39]	4.08	17 (68)			Kept
Needle aspiration [31]	4.04	72 (18)			Kept
Biopsy [34,39]	3.92	17 (68)	4.20	21 (84)	Kept
Skin graft [25,39,44]	3.92	17 (68)	4.08	18 (72)	Kept
Metalloproteinases [31]	3.76	17 (68)	3.80	17 (68)	Removed
Intermittent pneumatic compression [39]	3.76	15 (60)	3.44	14 (56)	Removed
Ultrasound therapy [39,44]	3.56	14 (56)	3.28	13 (52)	Removed
Hyperbaric oxygen therapy [39]	3.44	48 (12)			Removed
Near-infrared light therapy [39]	3.32	44 (11)			Removed
Electromagnetic therapy [39]	3.32	48 (12)			Removed

**Table 4:** Items related to quality of life measurement and health education.

Quality of Life Measurement					
	Mean Round 1	Exp ≥ 4 *n* (%)	Mean Round 2	Exp ≥ 4 *n* (%)	Final Decision
Chronic Venous Insufficiency Quality of Life Questionnaire (CIVIQ) [27]	4.76	23 (92)			Kept
Charing Cross [56]	4.64	22 (88)			Kept
EQ-5D [6,21,40,47]	4.40	20 (80)			Kept
RAND-36 [6,40,44,47,49]	3.96	18 (72)	3.72	14 (56)	Removed
Aberdeen Varicose Vein Questionnaire (AVVQ) [21,40]	3.88	18 (72)	3.88	16 (64)	Removed
**Health Education**					
Exercise regularly [29,33,34,42]	4.96	25 (100)			Kept
Keep mobile [33,42]	4.92	25 (100)			Kept
Implement nutritional interventions/ weight loss [30,31,33,34,42]	4.88	25 (100)			Kept
Use compression stockings [30,31,34,42]	4.88	25 (100)			Kept
Elevate legs when resting [29,30,31,33,34,42,51]	4.84	25 (100)			Kept
Avoid hot temperatures such as sitting too close to the fire [30,31,33,42]	4.80	25 (100)			Kept
Keep legs raised at night [30,31,33]	4.80	24 (96)			Kept
Wear well-fitted shoes [30,31,42]	4.80	24 (96)			Kept
Avoid tight clothing [30,31,33,42]	4.72	24 (96)			Kept
Shower as usual [30,33,42]	4.68	22 (88)			Kept
Prevent constipation [31,33,42]	4.60	24 (96)			Kept
Quit smoking [29]	4.60	21 (84)			Kept
Leg massage [29]	4.44	23 (92)			Kept
Use hyperoxygenated fatty acids [42]	4.28	21 (84)			Kept
Take cold showers [30]	4.28	20 (80)			Kept
Moisturize skin on legs [29,30,31,33,42]	4.60	24 (96)			Kept
